# Effects of cysteine and ascorbic acid in freezing extender on sperm characteristics and level of enzymes in post‐thawed stallion semen

**DOI:** 10.1002/vms3.315

**Published:** 2020-06-30

**Authors:** Mohaammed S. Alamaary, Abd W. Haron, Mark W. H. Hiew, Mohamed Ali

**Affiliations:** ^1^ Department of Veterinary Clinical Studies Faculty of Veterinary Medicine Universiti Putra Malaysia UPM Serdang Malaysia; ^2^ King Abdulaziz Arabian Horses Center Ministry of Environment Water Agriculture Riyadh Saudi Arabia; ^3^ Animal Production and Breeding Faculty of Agriculture and Veterinary Medicine Qassim University Qassim Saudi Arabia

**Keywords:** Arabian stallion, ascorbic acid, cysteine, enzymes, fertility prediction, frozen semen

## Abstract

Present study aimed to investigate the effect of adding antioxidants, cysteine and ascorbic acid on the levels of glutamic oxaloacetic transaminase (GOT), glutamic‐pyruvate (GPT), alkaline phosphatase (ALP), lactate dehydrogenase (LDH) and γ‐glutamyl transpeptidase (GGT) enzymes of post‐thawed stallion sperm. Ten ejaculates were collected each from four healthy stallions and cryopreserved using HF‐20 freezing extender containing either 0 mg/ml cysteine or ascorbic acid, 0.5 mg/ml cysteine and 0.5 mg/ml ascorbic acid. All samples in freezing extender containing cysteine or ascorbic acid or none of them were assessed for sperm motility, viability, plasma membrane integrity, morphology and enzymes concentration. The ALP, LDH and GGT were significantly higher in 0‐group compared with cysteine and ascorbic acid groups. The sperm motility of frozen‐thawed semen with 0‐group was significantly better compared with cysteine and ascorbic acid groups. The variation on viability, sperm membrane integrity and morphology were insignificant between all treated groups. Therefore, these enzymes were reduced when using antioxidants in the freezing extender. Results of the present study suggest that concentration of ALP, LDH and GGT enzymes could be used as parameters for prediction of frozen‐thawed stallion semen.

## Abstract 2

This manuscript aims to evaluate the effect of cysteine and ascorbic acid to HF‐20 extender on the frozen semen enzyme and using these enzymes as markers of frozen semen quality in stallion's semen. It shows that the level of ALP, LDH and GGT enzyme steadily correlated with post‐thawed semen quality. The samples treated with cysteine and ascorbic acid showed a deleterious effect of sperm enzyme concentration and function compared with control group.

## 1 INTRODUCTION

Lately, with the increase in the use of artificial insemination and shipped semen to most farms, evaluation of the semen quality has become an essential aspect to predict the performance of the semen to be used. In vitro assays have a limited predictability of semen fertility, and there is no single assay that correlates consistently with the fertility (Mocé & Graham, 2008). Thus, the increase in laboratory assays could improve the accuracy of fertility prediction. Furthermore, the spermatozoa function and structure could be damaged by the reactive oxygen species (ROS) reaction with the spermatozoa enzymes, membrane phospholipid and chromatin (Aitken, 2017). This causes lipid peroxidation, protein oxidation and DNA fragmentation (Bui, Sharma, Henkel, & Agarwal, 2018; Morielli & O’Flaherty, 2015; Treulen, Elena, Aguila, Uribe, & Felmer, 2018). In addition, cryopreservation technique revealed a high susceptibility to the excessive ROS production and the disruption on antioxidant levels and function in post‐thawed semen samples (Ball, 2008). Thus, balancing the ROS by adding antioxidants to the semen extender showed effective improvement to the semen preservation especially when the seminal plasma removed during semen preservation (Agarwal, Nallella, Allamaneni, & Said, 2004).

Various enzymes in the semen were reported to have an effect on sperm fertility (Pesch, Bergmann, & Bostedt, 2006). The level of these enzymes is affected by sperm membrane damage (Katila, 2001). Therefore, assessment of the concentration of the enzymes in the fresh semen sample has been used for a long time. Glutamic oxaloacetic transaminase (GOT) and glutamic‐pyruvate (GPT) are intracellular enzymes found in sperm cytoplasmic droplets (Katila, 2001). The alkaline phosphatase (ALP) enzyme is also isolated from the sperm plasma membrane (Bucci et al., 2014). Furthermore, the lactate dehydrogenase (LDH) enzyme is found in the cytosol, mitochondria and on the sperm plasma membrane (O’Flaherty, Breininger, Beorlegui, & Beconi, 2005). It is well known that γ‐glutamyl transpeptidase (GGT) enzyme is located on the external site of spermatozoa (Agarwal & Vanha‐Perttula, 1988). In addition, these enzymes play an essential role in sperm maturation and function (Pesch et al., 2006).

Cysteine is a semi‐essential amino acid that acts as an intracellular antioxidant and a main component of the antioxidant glutathione GSH. Therefore, cysteine is essential to balance the intracellular redox. Cysteine also plays a major role in protein function (Paul, Sbodio, & Snyder, 2018). Ascorbic acid or Vitamin C is a safe and water soluble antioxidant. Ascorbic acid is able to break chain reactions and remove free radicals during chain reactions to stop peroxidation from happening (Zhang, Yi, Chen, Hou, & Zhou, 2012). In addition, it can act as a co‐antioxidant that prevents lipid peroxidation. Ascorbic acid is an effective free radical scavenger due to its production of monodehydroascorbate (MDHA) radicals that are unable to react with other molecules or oxygen to produce more reactive radicals (Du, Cullen, & Buettner, 2012).

Therefore, the current study aimed to determine the effect of adding cysteine and ascorbic acid in the freezing extender on physical and biological characteristics, as well as the level of enzymes of post‐thawed frozen stallion sperm. There is possibility of using these enzymes as markers of frozen semen quality with the other sperm parameters, such as sperm motility pattern, viability, sperm membrane integrity and morphological defects.

## 2 MATERIALS AND METHODS

This study was exempted from approval from the Institution Animal Ethics because the semen collection using artificial vagina does not affect the normal physiology of the animals.

### 2.1 Animals and semen collection

Four healthy stallions, aged 4 to 14 years, were selected for the study, after a breeding soundness examination. All animals were housed individually in the King Abdulaziz Arabian Horse Center in Dirab (KAAH), which is a member of World Arabian Horse Organization (WAHO). They were fed pellets supplemented with Hijazi clovers three meals a day. Rhodes grass, mineral blocks and water were provided ad libitum. Epididymal sperm reserves were decreased by daily semen collection from all the stallions for 3 days. Afterwards, constant semen collections were performed using automated semen collection phantom (Equidame® phantom Haico‐Finland), twice a week per stallion. As such, a total of 10 ejaculates were collected for the study. The semen samples were collected during the winter from February to March.

### 2.2 Semen processing

The semen gel portion was removed immediately after collection, using sterile gauze and then transferred to a 37°C water bath. The semen volume was measured in a graduated cylinder. The ejaculate was also evaluated for total motility, progressive motility and sperm concentration. Sperm concentration and motility were determined using the CASA system (ISAS® program, Prosser R + D, Paterna, Valencia, Spain). In this light, samples with a minimum of 200 × 10^6^ sperm/ml and motility >60% were used for this study.

Filtered semen of each ejaculate was diluted (1:1) with a centrifugation media and then divided into three aliquots. Using a centrifuge at 800 g for 10 min, seminal plasma was removed and each tube of the sample was resuspended with HF‐20 containing either none (control) or 0.5 mg/ml cysteine, or 0.5 mg/ml ascorbic acid. The final semen concentration after dilution was 200 × 10^6^ sperm/ml. The sperm concentration was adjusted using a haemocytometer (Neubauer improved bright‐line, Germany). All the tubes were cooled to 4°C for 90 min, with the cooled semen being filled into 0.5 ml straws. The straws were further frozen by a programmable freezer (Automatic Freezer with Windows®‐tablet, 230 V, Minitube, Germany) (60°C/min. to –140°C).

### 2.3 Extenders

The centrifugation media were a mixture of 6.0 g glucose, 0.37 g ethylene‐diamine‐tetra‐acetic acid (EDTA), 0.37 g sodium citrate, 0.12 g sodium bicarbonate, 100,000 IU penicillin and 0.08 g streptomycin in 100 ml of distilled water. Freezing extender (HF‐20) contained 5 g glucose, 0.3 g lactose, 0.3 g raffinose, 0.15 g sodium citrate, 0.05 g sodium phosphate, 0.05 g potassium sodium tartrate, 10% egg yolk, 25,000 IU penicillin, 0.08 g streptomycin, 3% glycerol and deionized water up to 100 ml (Nishikawa, 1975).

### 2.4 Semen evaluation

Water bath at 37ºC was used to thaw the frozen straws for 30 s, with the contents being emptied into a small warmed tube. Evaluation of total and progressive motility was done using the ISAS® program. The samples were then assessed for plasma membrane integrity, morphology defects, viability and enzyme levels.

### 2.5 Assessment of sperm motility

The ISAS® program (CASA system) was used to assess the motility pattern immediately after the dilution of the semen or post‐thawed semen. A sample (2.7 μl) from each tube was placed on a slide (ISASD4C, Prosser R + D, Paterna, Valencia, Spain) and semen motility assessment was performed based on five digital images from different fields, via a × 10 negative‐phase contrast objective and warm stage at 37ºC. The motility pattern was measured according to total motile sperms (TMS %), rapid progressively sperm (RPS %), curvilinear velocity (VCL μm/s), straight line velocity (VSL μm/s), average path velocity (VAP μm/s), linearity index (LIN %) and straightness index (STR %). At least 300 sperm were analysed from each sample, with the images turning read within 1 s. Five images from different fields for each sample were analysed.

### 2.6 Plasma membrane integrity

The hypo‐osmotic swelling test (HOST) was used to assess the plasma membrane integrity of spermatozoa. A minimum of 200 sperm were analysed for coiled tail, using phase contrast microscopy (×400). A mixture of a sucrose‐based solution 1,000 μl and 20 μl of semen at 100 mOsmol was incubated at 37°C for 50 min in a water bath (Neild et al., 1999).

### 2.7 Viability

Sperm viability was evaluated using acridine orange (AO) and propidium iodide (PI). This kit was purchased from Halotech DNA S.L. Spain. Firstly, the semen was diluted to 10–15 × 10^6^ sperm/ml. The sperm concentration was adjusted using a haemocytometer (Neubauer improved bright‐line, Germany). Afterwards, 10 μl of diluted semen was placed on a slide. The next step implied mixing 1.0 μl of AO and PI with the diluted semen. Finally, the mixture was covered and evaluated with a fluorescence microscope. Live sperm retained the AO, giving green fluorescence, while PI penetrated the damaged sperm, causing red fluorescence. A total of 300 sperm were assessed per sample.

### 2.8 Morphology

Sperm morphology was examined using the eosin–nigrosin staining technique (RAL Diagnostics, Martillac, France). A drop of semen (15 μl) was mixed with 15 μl of eosin–nigrosin on a clean and warm slide and smeared. The mixture was gently spread and evaluated randomly under oil immersion at 1,000 × magnification. Spermatozoa morphological defects that were recorded normal, abnormal acrosome, abnormal head, abnormal midpiece, abnormal tail, head detached, proximal droplet, distal droplet, bent tail and others. The sperm morphology was classified as normal, major abnormalities and minor abnormalities. (Murcia‐Robayo, Jouanisson, Beauchamp, & Diaw, 2018).

### 2.9 Enzymes

The enzymes were assessed using Humalyzer 3,000, and liquid reagents from Tufnell Drive, Kamwokya, Kampala and Uganda were used for each enzyme. All steps were done following the manufacturer instructions. Two straws (1 ml) from each extender were thawed in the water bath at 37°C for 30 s. The thawed semen was centrifuged at 2500 g for 12 min. Then, the supernatant was mixed with normal saline (1:11). The standard sample was distilled water, which was analysed in the Humalyzer system before introducing the samples. The liquid reagents were prepared following the kit instructions and mixed with sperm extracts in the ratio of 1:10, i.e. 100 μl of sperm extracts was mixed with 1,000 μl of prepared solution. The enzymes were measured in U/L.

### 2.10 Statistical Analysis

The one‐way ANOVA test for comparison was used to analyse the data. The analysis was considered significant at *p* < .05. SPSS statistical software version 16.0 was used to analyse the data.

### 2.11 Data availability statement

The data that support the findings of this study are available from the corresponding author upon reasonable request.

## 3 RESULTS

### 3.1 Motility

The result of sperm motility of frozen‐thawed semen with HF‐20 (0), 0.5 mg/ml cysteine and 0.5 mg/ml ascorbic acid is illustrated in Table 1. The differences among HF‐20 (0) and 0.5 mg/ml cysteine on the total motility were not significant (*p* > .05), but there was a significantly better result on the HF‐20 (0) as compared to 0.5 mg/ml ascorbic acid. HF‐20 (0) showed better RPS, VCL, VSL and VAP (*p* > .05). Ascorbic acid 0.5 mg/ml showed the lowest result (*p* > .05) on VSL, as compared with the other groups.

**Table 1:** The motility pattern on the groups of HF‐20 extenders (0, 0.5 cysteine and 0.5 ascorbic acid) on stallion frozen semen

Parameters	Extenders		
	HF−20 (0)	Cysteine 0.5 mg/ml	Ascorbic acid 0.5 mg/ml
TMS (%)	44.99 ± 4.72^a^	37.21 ± 5.84^ab^	28.75 ± 5.23^b^
RPS (%)	22.09 ± 2.16^a^	10.01 ± 3.19^b^	7.70 ± 3.22^b^
VCL (µm/s)	106.53 ± 2.90^a^	86.76 ± 1.43^b^	83.22 ± 2.18^b^
VSL (µm/s)	32.48 ± 1.02^a^	26.72 ± 1.58^b^	21.46 ± 1.29^c^
VAP (µm/s)	60.57 ± 0.43^a^	44.39 ± 0.63^b^	42.98 ± 1.20^b^
LIN (%)	31.30 ± 1.49^ab^	31.70 ± 1.97^a^	26.76 ± 1.33^b^
STR (%)	53.60 ± 1.68^ab^	59.51 ± 2.88^a^	49.96 ± 2.02^b^

Note

Abbreviations: LIN, Linearity index; RPS, Rapid Progressive Sperm; STR, Straightness index; TMS, total motile sperms; VAP, Average Value; VCL, Curvilinear speed; VSL, Rectilinear speed.

All values were expressed as mean ± SE.

^ab^ Values with different superscripts across rows indicate significant differences at *p* < .05.

### 3.2 Membrane Integrity, Viability and morphology

Sperm membrane integrity, viability and morphology defects are shown in Table 2. The results of sperm membrane integrity, viability and sperm morphology revealed no significant variation among all extenders.

**Table 2:** The plasma membrane integrity, viability and morphology on the groups of HF‐20 extenders (0, 0.5 cysteine and 0.5 ascorbic acid) on stallion frozen semen

Parameters	Extenders		
	HF−20 (0)	Cysteine 0.5 mg/ml	Ascorbic acid 0.5 mg/ml
HOST (%)	39.89 ± 9.56	27.94 ± 7.03	21.77 ± 4.69
Viability (%)	32.40 ± 8.24	30.56 ± 9.50	19.73 ± 2.27
Normal Morphology (%)	80.34 ± 3.23	83.87 ± 0.83	83.66 ± 2.45
Major Abnormalities (%)	7.40 ± 4.61	4.43 ± 0.42	5.65 ± 1.72
Minor Abnormalities (%)	11.78 ± 1.47	11.88 ± 1.10	14.27 ± 2.55

Note

Abbreviation: HOST, Hypo‐osmotic swilling test (sperm membrane integrity).

All values were expressed as mean ± SE. No significant differences between groups at *p* <.05.

### 3.3 Enzymes

Table 3 reveals the result of enzymes levels in post‐thawed semen in HF‐20 (0), 0.5 mg/ml cysteine and 0.5 mg/ml ascorbic acid groups. The enzymes GOT, ALP, LDH and GGT were significantly (*p* > .05) higher in HF‐20 (0) when compared with groups treated with 0.5 mg/ml cysteine or ascorbic acid. The variation between HF‐20 (0) and 0.5 mg/ml ascorbic acid groups on LDH concentration was insignificant. Furthermore, no significant (*p* > .05) differences were observed between cysteine 0.5 mg/ml and ascorbic acid 0.5 mg/ml groups for earlier mentioned enzymes. The concentration of GPT enzyme did not show any variation (*p* > .05) among all extenders.

**Table 3:** The level of enzymes in HF‐20 (0), 0.5 cysteine and 0.5 ascorbic acid in horses frozen semen

Enzymes	Extenders		
	HF−20 (0)	Cysteine 0.5 mg/ml	Ascorbic acid 0.5 mg/ml
GPT U/L	48.03 ± 14.31^a^	56.83 ± 17.18^a^	39.23 ± 11.73^a^
GOT U/L	146.30 ± 52.60^a^	27.86 ± 7.4^b^	35.20 ± 13.26^b^
ALP U/L	2,477.20 ± 448.7^a^	1,322.56 ± 58.8^b^	679.80 ± 71.52^b^
LDH U/L	809.23 ± 178.5^a^	384.2 ± 76.9^b^	466.03 ± 12.6^ab^
GGT U/L	410.30 ± 29.2^a^	248.96 ± 8.09^b^	292.60 ± 1.6^b^

Abbreviations: ALP, Alkaline phosphatase; GGT, γ‐glutamyl‐transferase; GOT, glutamic‐oxaloacetic transaminase; GPT, glutamic‐pyruvate; LDH, Lactate dehydrogenase.

^abc^ Values with different superscripts across rows indicate significant differences (*p* < .05).

## 4 DISCUSSION

Although the routine examination of semen quality such as sperm concentration, motility, morphology and viability improves the fertility prediction, the reliability of these assays is limited. Moreover, the pregnancy rate itself is still the best indicator of fertility of stallions (Varner, 2016). The level of alkaline phosphatase (ALP), lactate dehydrogenase (LDH) and γ‐glutamyl transpeptidase (GGT) enzymes in the seminal plasma is positively correlated with sperm function and semen quality (Bucci et al., 2014; Milinkovi, 2016).

Glutamic‐oxaloacetic (GOT) and glutamic‐pyruvate (GPT) are intracellular enzymes and, for a long time, these enzymes were used as markers for sperm membrane damage. Leakage of these enzymes into the seminal plasma is a sign of damage to the sperm membrane (Katila, 2001; Tuli & Singh, 1982). However, the use of GOT and GPT enzymes as an indicator of frozen semen quality in previous studies showed contradictory results. In the current study, using post‐thawed stallion spermatozoa extract, a high concentration of GOT in HF‐20 (0) was observed that showed higher sperm quality as compared to 0.5 mg/ml cysteine and 0.5 mg/ml ascorbic acid. Furthermore, the GPT enzyme level did not reveal any significant difference among all extenders. This result is in agreement with the study by Purdy (2006) and Tuli, Schmidt‐Baulain, and Holtz (1991), conducted on goats, and in disagreement with the study by Tuli and Singh (1982). The reason for this result could be due to the presence of GOT and GPT in cytoplasmic droplets (Katila, 2001). Therefore, the reliability and validity of these enzymes as markers of semen quality is lower than the other enzymes. Furthermore, adding cysteine and ascorbic acid in this study adversely affect the sperm GOT enzyme level.

Alkaline phosphatase enzyme (ALP) showed higher concentration on HF‐20 (0) extender when compared with cysteine 0.5 mg/ml and ascorbic acid 0.5 mg/ml. The high level of ALP was positive with sperm total, progressive motility and other spermatozoa motility pattern on HF‐20 (0). This result is in agreement with other findings in humans (Alibawi, Al‐morshidy, & Alhuweizi, 2012), canine (Frenette, Dubé, & Tremblay, 1986) and horses ( Turner & McDonnell, 2003), which revealed positive correlations between (ALP) concentration and semen quality in the sperm or semen plasma of fresh semen. Kareskoski et al. (2010) reported high correlation between the sperm concentration and ALP level in the stallion's seminal plasma using fresh semen. The results of the present study also emphasized an interrelationship of post‐thawed semen motility and viability with the level of ALP (Bucci et al., 2016). Therefore, the level of ALP enzyme could be a sign of frozen semen quality in the Arabian stallion. Furthermore, cysteine and ascorbic acid did not protect the sperm during cryopreservation and affect adversely on the sperm motility and ALP concentration.

Dogan, Polat, and Nur (2009) and Pesch et al. (2006a) claimed that lactate dehydrogenase (LDH) and glutamyl‐transferase (GGT) enzymes are accurate parameters for semen quality on horse fresh semen. Moreover, a high concentration of glutamyl‐transferase (GGT) and lactate dehydrogenase (LDH) was found on frozen stallion semen, which was corresponding to the high sperm motility. These findings are consistent with Pero et al. (2017) who reported a high concentration of GGT with higher sperm motility on frozen bovine semen. In addition, a significantly higher LDH and GGT concentration on frozen stallion semen extract can be observed in the present result. The LDH and GGT concentration were higher in the control groups than groups treated with either cysteine or ascorbic acid. These results could be due to the correlation between (LDH) level and mitochondria, and sperm plasma membrane integrity. Laudat, Foucault, and Palluel (1997) found LDH enzyme in the cytosol, mitochondria and on the sperm plasma membrane.

The high concentration of ALP, LDH and GGT enzymes on frozen spermatozoa was corresponding to arise sperm motility pattern, whereas the other parameters, such as viability, sperm membrane integrity and morphological defects did not reveal any variation with the different extenders. Evaluating these enzymes seems to be more effective on post‐thawed semen evaluation in the stallion. Even though the assessment of the viability and sperm membrane integrity showed a better result in HF‐20 (0), as compared to 0.5 mg/ml cysteine and 0.5 mg/ml ascorbic acid, the differences were not significant. These results agreed with the previous studies conducted by Dogan et al. (2009), Pero et al. (2017) and Pesch et al. (2006b). The level of these enzymes seemed to have a positive relationship with spermatozoa metabolism and other functions. The antioxidants cysteine and ascorbic acid were used to protect the sperm during cryopreservation procedure and encountering the excessive ROS. In this study, adding cysteine and ascorbic acid to the freezing extender excess the acidity of extender (lower pH) that was affecting negatively the enzymes level and the motility of sperm.

The intracellular enzymes, GOT and GPT, are known as mediators of spermatozoa metabolism activities, which are important for sperm motility and fertility (Dogan et al., 2009). The addition of either cysteine or ascorbic acid in the current study reduced the GOT enzyme concentration in stallion's post‐thawed semen. The decrease in the GOT enzyme corresponded to the poor sperm motility in this result, which can be a consequence of the disruption on the sperm's metabolism. These findings agree with the study conducted on goats, performed by Purdy (2006), but contradict those performed on pigs (Eugenia et al., 2013), which concluded that a reduction in sperm motility occurred with a high concentration of GOT enzyme in the seminal plasma. It has been noted that an effect of Vitamin B6 pyridoxal‐5' phosphate concentration on the activity of GOT enzyme in bull, ram and boar seminal plasmas was correlated with sperm concentration and motility (Ciereszko, Glogowski, Demianowicz, & Strzezek, 1994). This could explain the contradiction in semen quality with the variance in GOT enzyme concentrations. On the other hand, the GPT enzyme concentration was not affected by the addition of either cysteine or ascorbic acid, which is in line with findings of Roychoudhury, Pareek, and Gowda (1974). A particular research work (Tuli & Singh, 1982) found differences between the GOT and GPT enzyme concentrations from the same sample using different frozen semen extenders, which can be a result of the effect of substances used in the semen extender with these enzymes levels.

Alkaline phosphatase (ALP) is an enzyme, which was reported to be able to prevent the premature spermatozoa capacitation that improves the sperm fertility. In addition, ALP affects the spermatozoa metabolism rates (Bucci et al., 2013). Frozen semen showed higher vulnerability to premature capacitation than fresh semen, which was associated with the decrease in ALP activity (Bucci et al., 2016). The current results revealed higher ALP concentration in the HF‐20 (0) compared to 0.5 cysteine and 0.5 ascorbic acid groups. The decrease in ALP levels was correlated with the sperm motility in the groups treated with cysteine or ascorbic acid, which can generate from the disruption in the sperm metabolism. Furthermore, the sample treated with either cysteine or ascorbic acid might be more susceptible to premature capacitation that reduced the sperm fertility. As such, the deleterious effect of these antioxidants on the ALP levels and activity can lead to the above situation.

On the other hand, the high levels of lactate dehydrogenase (LDH) and sperm motility in semen cryopreserved with HF‐20 (0) extenders might be due to the characteristics of this enzyme type, which generates the energy required to enhance the sperm motility feature. Furthermore, LDH enzymes were reported to play a role in sperm capacitation by providing nicotinamide adenine dinucleotide (NADH) that is necessary for increasing the sperm's capability (O’Flaherty, Beorlegui, & Beconi, 2002). It has also been reported that LDH enzyme is an essential enzyme for sperm acrosome reaction (O’Flaherty et al., 2005). Therefore, despite the fact that the higher LDH enzyme level, the better the semen quality, this enzyme could be supplemented to the semen extender to improve its quality. The addition of the antioxidants in this result, i.e. either cysteine or ascorbic acid, adversely affects the LDH enzyme activity. Thus, it can have an effect on the sperm capability and acrosome reaction as well.

Gamma‐glutamyl‐transferase GGT enzyme activities revealed a high correlation with the sperm's progressive motility (Stefanov et al., 2013). This can be a result of the enzyme's ability to enhance the glutathione metabolism and protect the sperm during freezing procedures. Increased glutathione concentration was also observed in the presence of the GGT enzyme in previous study (Seligman, Newton, Fahey, Shalgi, & Kosower, 2005). Moreover, GGT acted as a mediator for sperm thiol oxidation that improved the spermatozoa cryosurvival and sperm capacitation (Kaneko, Whittingham, Overstreet, & Yanagimachi, 2003). It was also observed that GGT enzymes play a role in controlling ROS in the semen. The present study has shown an adverse effect with regards to adding cysteine and ascorbic acid in correlation with the GGT enzyme concentration. This could be the reason which contributed to poor sperm motility and the reduction in the protection of spermatozoa during the freezing procedures in extenders supplemented with cysteine or ascorbic acid, compared with the HF‐20 (0) groups. Furthermore, the disruption towards the GGT enzyme might be followed by a disruption towards the oxidative stress level on the semen samples.

## 5 CONCLUSIONS

To conclude with, using alkaline phosphatase (ALP), lactate dehydrogenase (LDH) and γ‐glutamyl‐transferase (GGT) enzymes as markers of sperm quality is a positive sign in predicting the characteristics of post‐thawed stallion's semen. In contrast, glutamic‐pyruvate (GPT) and glutamic‐oxaloacetic transaminase (GOT) showed limited benefits in semen evaluation in the current study.

In addition, supplementing cysteine or ascorbic acid in this study decreased the ALP, LDH, GGT and GOT enzymes concentrations in the frozen semen. As such, this could have led to the poor post‐thawed semen quality in the extenders, which were treated, with either cysteine or ascorbic acid. The addition of cysteine or ascorbic acid did not show any benefit to the frozen semen extender in the stallion.

## CONFLICT OF INTEREST

We declare that there is no conflict of interest in the research.

## AUTHOR CONTRIBUTIONS

Alamaary Mohammed Saad, Abd Wahid Haron, Mark Hiew Wen and Mohamed Ali conceived the idea and designed the main frame of this manuscript as part of Alamaary Mohammed Saad's research work under the supervision of Abd Wahid Haron.
